# The Value of Contrast-Enhanced Ultrasonography and Contrast-Enhanced CT in the Diagnosis of Malignant Renal Cystic Lesions: A Meta-Analysis

**DOI:** 10.1371/journal.pone.0155857

**Published:** 2016-05-20

**Authors:** Dong Lan, Hong-Chen Qu, Ning Li, Xing-Wang Zhu, Yi-Li Liu, Chun-Lai Liu

**Affiliations:** 1 Department of Urological Surgery, The Fourth Affiliated Hospital of China Medical University, Shenyang, Liaoning, P.R. China; 2 Department of Urological Surgery, Cancer Hospital of China Medical University/Liaoning Cancer Hospital & Institute, Shenyang, Liaoning, P.R. China; ACTREC (Advanced Centre for Treatment, Research and Education in Cancer) / Tata Memorial Centre, INDIA

## Abstract

We compared the efficacy of contrast-enhanced ultrasound (CEUS) and contrast-enhanced computed tomography (CECT) for the diagnosis of renal cystic lesions via a meta-analysis to determine the value of CEUS in the prediction of the malignant potential of complex renal cysts. Eleven studies were evaluated: 4 control studies related to CEUS and CECT, 3 studies related to CEUS and 4 studies related to CECT. According to the random effects model, the pooled sensitivity, specificity, positive likelihood ratio, and negative likelihood ratio for CEUS/CECT were 0.95/0.90, 0.79/0.85, 4.39/5.00, and 0.10/0.15, respectively. The areas under the summary receiver operating characteristic (AUCs-SROC) curves for the two methods were 94.24% and 93.39%, and the estimated Q values were 0.8805 and 0.8698, respectively. Comparing the Q index values of CEUS and CECT revealed no significant difference between the two methods (P>0.05). When compared with conventional CECT, CEUS is also useful for diagnosing renal cystic lesions in the clinic.

## Introduction

Renal-occupying lesions include cystic and solid masses that are usually found incidentally during an imaging examination. Most of these lesions are simple benign cysts that can be clearly diagnosed by color Doppler ultrasound, and solid masses can also be diagnosed confidently by contrast-enhanced computed tomography (CECT) because of their significant blood supply. However, some benign lesions, such as hemorrhagic cysts, inflammatory cysts and multiple cystic renal tumors, have a complex appearance on CT that makes it difficult to distinguish malignant cysts, such as cystic renal cell carcinomas. As the prognoses of benign complicated cysts and cystic renal cell carcinomas can be completely different, the acquisition of a definitive diagnosis is very important. Contrast-enhanced ultrasound (CEUS) has been used in the diagnosis and differential diagnosis of renal cystic lesions because of its effective visualization of the wall, septa, solid components and blood supply [1]. Substantial research regarding the use of CECT and CEUS in the diagnosis of renal cystic lesions has been performed in recent years. However, different diagnostic efficiencies of the two methods have been reported. Therefore, we examined the relevant literature regarding the diagnosis of renal cystic lesions related to CEUS and CECT and drew conclusions from the results of a meta-analysis, which may provide a basis for clinical diagnosis.

## Materials and Methods

We performed an electronic search of the Cochrane Library, PubMed, Embase, and Ovid databases to identify relevant articles published in the last 15 years (January 1, 2000 to April 30, 2015). The search terms included cystic, renal mass, kidney cyst, contrast-enhanced ultrasound, ultrasound (US), contrast-enhanced CT, and computed tomography, and the search strategy included the Bayes Library of Diagnostic Study and Reviews. We then utilized a combination of subject words and free words and adjusted the search method according to the specific database. Two reviewers(Dong Lan, Hong-Chen Qu) searched the literature independently, and references in eligible textbooks were also reviewed.

### 1. Inclusion and exclusion criteria

#### 1.1 Patient examination

CEUS: In all of the patients of the included studies, CEUS examination was performed after conventional US examination using the same scanning technique.

CECT: All of the patients of the included studies underwent helical CT scanning in which unenhanced images were obtained first, followed by the administration of a contrast agent and multi-phase delayed enhanced scanning to produce corticomedullary-phase, nephrographic-phase and excretory-phase images.

#### 1.2. Inclusion criteria

The inclusion criteria were as follows. (1) The gold standard applied for diagnosis was pathological diagnosis or follow-up observation. (2) The diagnostic test for renal cystic lesions in the study was related to CEUS or CECT technology. (3) The time interval between the results of the CEUS or CECT and pathological diagnosis was within 30 days. (4) The number of cases in the study was not less than 30 cases. (5) Four-grid data could be extracted from the data provided by the study.

#### 1.3 Exclusion criteria

The exclusion criteria were as follows. (1) The diagnostic test for renal cystic lesions in the study was only related to conventional ultrasonography or CT technology. (2) The literature consisted of conference papers or secondary literature, such as experience exchanges, abstracts, lectures and reviews.

### 2. Quality assessment of included studies

Two reviewers (Ning Li, Xing-Wang Zhu) independently evaluated the quality of the included literature, and discussion was necessary when they disagreed: according to QUADAS, items included "yes", "no", and "not clear", where "yes" means that the criteria are met, "no" means that the criteria are not met, and "not clear" indicates partial criteria satisfaction or that insufficient information was provided [2].

### 3. Statistical analysis

The true positive value (TP), true negative value (TN), false positive value (FP) and false negative value (FN) were extracted or calculated according to the original data of the included studies(Dong Lan, Hong-Chen Qu). The data were input into MetaDiSc version 1.4[3] using a heterogeneity test. The pooled sensitivity, specificity, positive likelihood ratio and negative likelihood ratio were calculated as well as the summary receiver operative characteristic (SROC) curve. As the area under the curve increased and the SROC curve shifted to the upper left corner, the value of the diagnostic test increased. P<0.05 indicated statistical significance. Two reviewers(Chun-Lai Liu, Yi-Li Liu) independently input the data into the statistical software programs and obtained the same results.

## Results

### 1. Characteristics of the included studies

**Fig 1:**
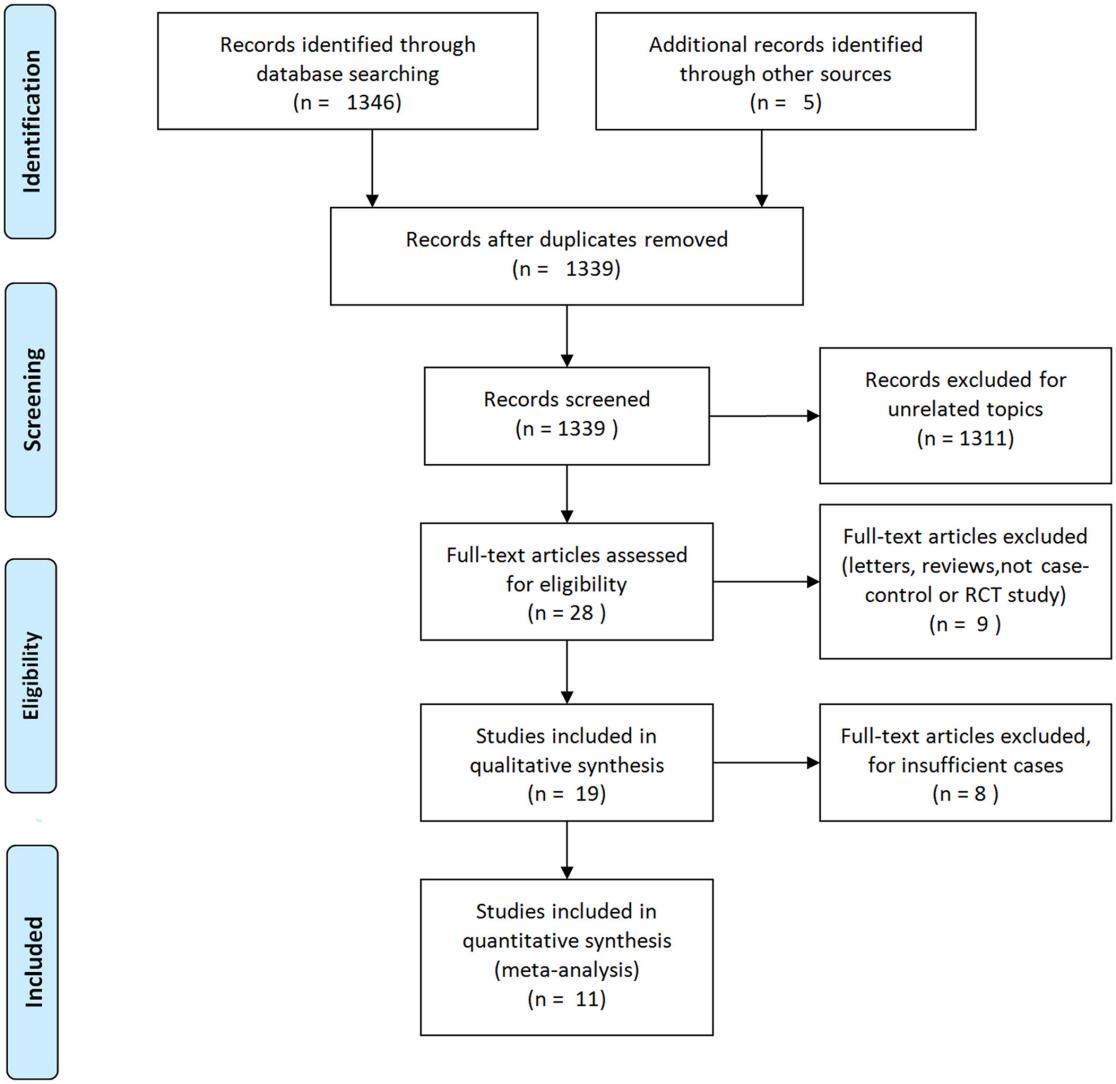
The flow chart shows the study selection procedure. In this meta-analysis, 11 studies were selected for qualitative analysis. Among these 11 studies, 444 cases were included for CEUS and 576 cases were included for CECT.

A total of 1339 studies were found in the primary search, and 28 studies were retained after primary screening according to the inclusion and exclusion criteria. After reading the full text, we identified five reviews; three studies of renal cystic lesions in which four-grid data could not be extracted; one study in which the data provided by the ultrasound examination were all positive cases, which hampers the calculation of some parameters; two studies that did not use the gold standard; two studies that included fewer than 30 cases; two studies that were only related to conventional ultrasound or CT technology; and two studies that used MRI. Finally, eleven manuscripts were found to be eligible, including four control studies related to both CEUS and CECT, three studies related to CEUS and four studies related to CECT. A total of 444 cases involving CEUS and 576 cases involving CECT were included in this study. The selection procedure used for the studies is shown in Fig 1.

The characteristics of the included literature are shown in Tables 1 and 2, and the quality evaluation of each study is shown in Table 3.

**Table 1:** The included studies related to CEUS.

Author	year	Region	TC^1^	TP	FP	FN	TN	PC^2^	FC^3^	Methods
*Y Xu*^[^^24^^]^	2014	China	87	63	14	0	10	87	0	REP^4^
*LY Xue*^[^^7^^]^	2014	China	103	54	7	4	38	103	0	REP
*Carlos*^[^^8^^]^	2014	Spain	62	10	3	1	48	12	50	REP, PRO^5^
*C Chen*^[^^9^^]^	2014	China	71	35	10	1	25	43	28	RCT
*DA Clevert*^[^^11^^]^	2008	German	37	10	7	1	19	14	23	REP, PRO
*Quaia E*^[^^10^^]^	2008	America	40	18	4	3	15	40	0	REP
*Ascenti*^[^^12^^]^	2007	Italy	44	6	4	0	34	9	35	REP, PRO

^1^ “TC” indicates total cases,

^2^”PC” indicates pathological cases,

^3^ “FC” indicates follow-up cases,

^4^ “REP” indicates a retrospective study,

^5^ “PRO” indicates a prospective study.

**Table 2:** The included studies related to CECT.

Author	year	Region	TC^1^	TP	FP	FN	TN	PC^2^	FC^3^	methods
*L Y*. *Xue*^[^^7^^]^	2014	China	70	29	5	6	30	70	0	REP^4^
*MH Kim*^[^^13^^]^	2014	Korea	164	52	17	6	89	164	0	REP
*DY* *Kim* ^[^^25^^]^	2010	Korea	125	48	8	5	64	125	0	REP
*Quaia E*^[^^10^^]^	2008	America	40	17	6	4	13	40	0	REP
*D*.*A Clevert*^[^^11^^]^	2008	German	37	10	5	1	21	14	23	REP, PRO^5^
*Ascenti*^[^^12^^]^	2007	Italy	44	6	4	0	34	9	35	REP, PRO
*Benjaminov*^[^^26^^]^	2006	America	32	21	6	0	5	32	0	REP
*Israel*^[^^23^^]^	2004	America	64	20	3	1	40	25	39	REP, PRO

^1^ “TC” indicates total cases,

^2^”PC” indicates pathological cases,

^3^ “FC” indicates follow-up cases,

^4^ “REP” indicates a retrospective study,

^5^ “PRO” indicates a prospective study.

**Table 3:** Quality evaluation of the included literature.

QUADAS items	Included reference										
	[7]	[8]	[9]	[10]	[11]	[12]	[13]	[23]	[24]	[25]	[26]
1. Was the spectrum of patients representative of the patients who will receive the test in practice?	Yes	Yes	Yes	Yes	Yes	Yes	Yes	Yes	Yes	Yes	Yes
2. Were selection criteria clearly described?	Yes	No	Yes	Yes	Yes	Yes	Yes	Yes	Yes	Yes	Yes
3. Is the reference standard likely to correctly classify the target condition?	Yes	Yes	Yes	Yes	Yes	Yes	Yes	Yes	Yes	Yes	Yes
4. Is the time period between the reference standard and index test short enough to be reasonably sure that the target condition did not change between the two tests?	Yes	Yes	Yes	Yes	Yes	Yes	Yes	Yes	Yes	Yes	Yes
5. Did the whole sample or a random selection of the sample receive verification using a reference diagnostic standard?	Yes	Yes	Yes	Yes	Yes	Yes	Yes	Yes	Yes	Yes	Yes
6. Did patients receive the same reference standard regardless of the index test result?	Yes	No	No	Yes	No	No	Yes	No	Yes	Yes	Yes
7. Was the reference standard independent of the index test (i.e., the index test did not form part of the reference standard)?	Yes	Yes	Yes	Yes	Yes	Yes	Yes	Yes	Yes	Yes	Yes
8. Was the execution of the index test described in sufficient detail to permit replication of the test?	Yes	Yes	Yes	Yes	Yes	Yes	Yes	Yes	Yes	Yes	Yes
9. Was the execution of the reference standard described in sufficient detail to permit its replication?	Yes	Yes	Yes	Yes	Yes	Yes	Yes	Yes	Yes	Yes	Yes
10. Were the index test results interpreted without knowledge of the results of the reference standard?	Yes	Yes	Yes	Yes	Yes	Yes	Yes	Yes	Yes	Yes	Yes
11. Were the reference standard results interpreted without knowledge of the results of the index test?	Yes	Yes	Yes	Yes	Yes	Yes	Yes	Yes	Yes	Yes	Yes
12. Were the same clinical data available when test results were interpreted as would be available when the test is used in practice?	Un*	Un	Un	Un	Un	Un	Un	Un	Un	Un	Un
13. Were intermediate test results reported?	Yes	Yes	Yes	Yes	Yes	Yes	Yes	Yes	Yes	Yes	Yes
14. Were withdrawals from the study explained?	Yes	Yes	Yes	Yes	Yes	Yes	Yes	Yes	Yes	Yes	Yes

* “Un” indicates “unclear**”**.

### 2. Analysis results

#### 2.1 Heterogeneity test

CEUS: The Spearman correlation coefficient of the logarithm of sensitivity and the logarithm of (1-specificity) was 0.541 (P = 0.210), which shows that there was no threshold effect. The Chi-square value of the pooled sensitivity was 11.13 (P>0.05), and the I-square was 46.1%, which showed that the data had moderate heterogeneity. The Chi-square value of the pooled specificity was 30.40 (P<0.05), and the I-square was 80.3%, which showed that the data had high heterogeneity. Therefore, we used a random effect model to analyze the data.

CECT: The Spearman correlation coefficient of the logarithm of sensitivity and the logarithm of (I-specificity) was -0.119 (P = 0.779), which shows that there was no threshold effect. The Chi-square values of the pooled sensitivity and pooled specificity were 9.72 (P>0.05) and 17.12 (P<0.05), respectively. The I-square values were 28.0% and 59.1%, respectively, indicating that the data had high heterogeneity. Thus, we used a random effect model to analyze the data.

#### 2.2 The results of the pooled analysis

The sensitivity, specificity, positive likelihood ratio, and negative likelihood ratio were calculated according to the TP, TN, FP and FN values, which were obtained for each study. The circles represent the point estimates of the sensitivity and specificity for each study. The circle size indicates the weight of the data, and the lines represent the corresponding range of the 95%Cis. The diamonds represent the pooled sensitivity and pooled specificity that were derived from the random effects model. The lines represent the corresponding range of the 95%Cis.

**Fig 2:**
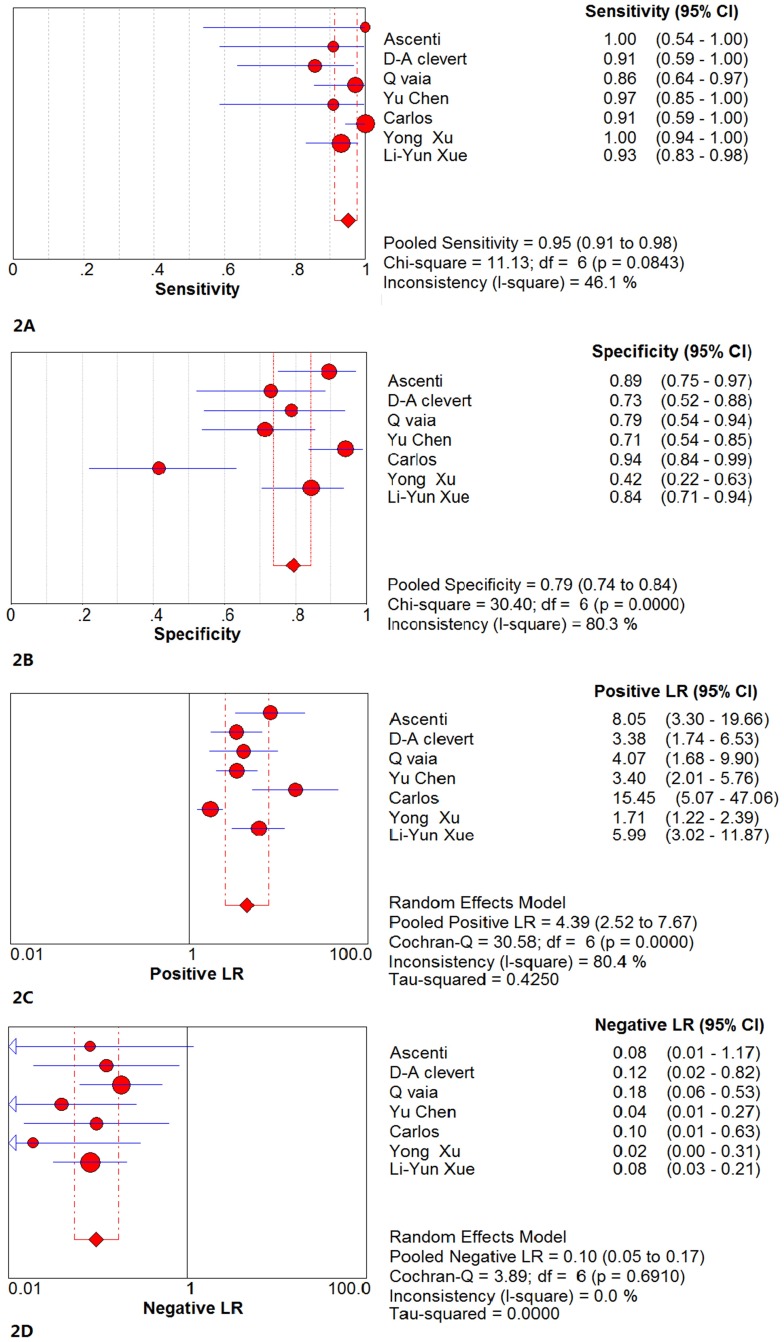
The forest chart of the pooled analysis results of CEUS. (2A)The forest chart of the pooled sensitivity of CEUS. The result of the pooled sensitivity was 0.95 (95%CI = 0.91~0.98). The Chi-square value of the pooled sensitivity was 11.13 (P>0.05), and the I-square was 46.1%. (2B)The forest chart of the pooled specificity of CEUS. The result of the pooled specificity was 0.79 (95%CI = 0.74~0.84). The Chi-square value of the pooled specificity was 30.40 (P<0.05), and the I-square was 80.3%. (2C)The forest chart of the pooled positive likelihood ratio of CEUS. The pooled positive likelihood ratio was 4.39 (95%CI = 2.52~7.67). (2D)The forest chart of the pooled negative likelihood ratio of CEUS. The pooled negative likelihood ratio was 0.10 (95%CI = 0.05~0.17).

CEUS: The results of the pooled sensitivity and pooled specificity with their 95%Cis were 0.95 (0.91~0.98) and 0.79 (0.74~0.84), respectively. The pooled positive likelihood ratio and the pooled negative likelihood ratio were 4.39 (2.52~7.67) and 0.10 (0.05~0.17), respectively. These values are shown in Fig 2.

**Fig 3:**
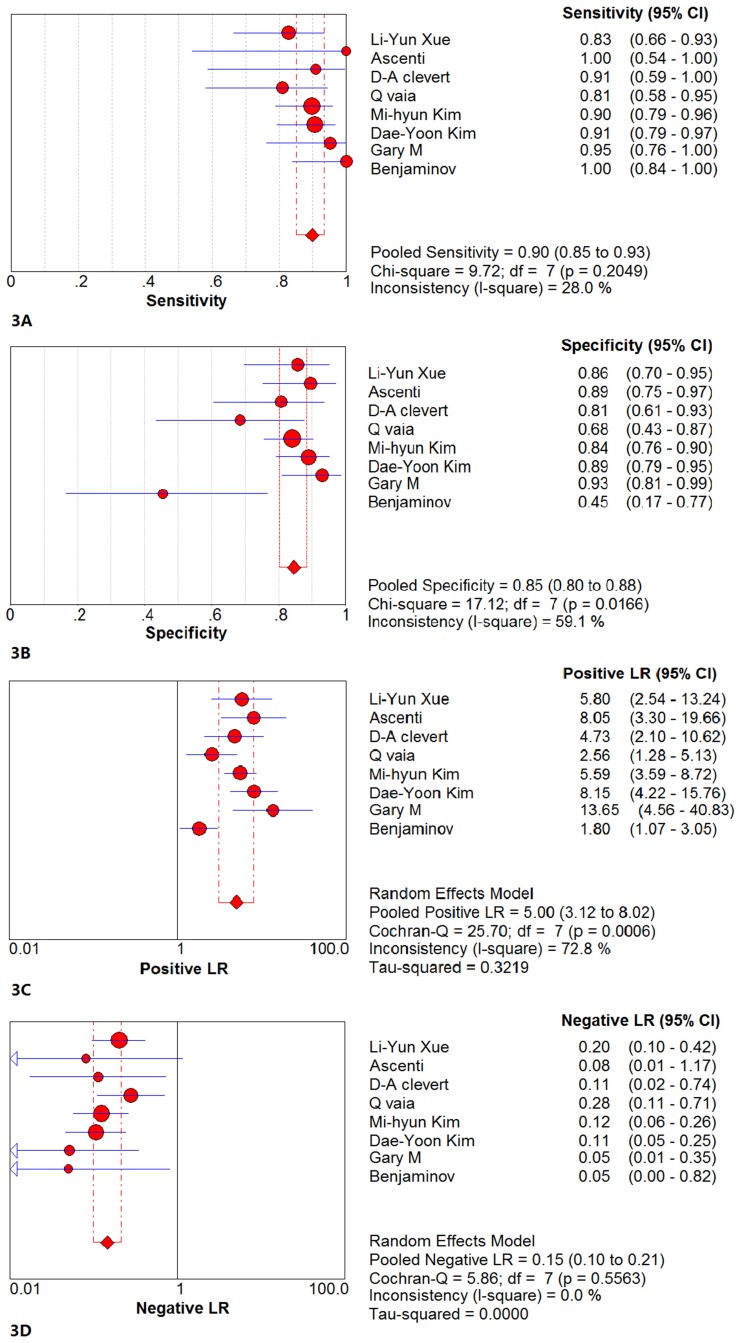
The forest chart of the pooled analysis results of CECT. (3A)The forest chart of the pooled sensitivity of CECT. The result of the pooled sensitivity was 0.90 (95%CI = 0.85~0.93). The Chi-square value of the pooled sensitivity was 9.72 (P>0.05), and the I-square was 28.0%. (3B)The forest chart of the pooled specificity of CECT. The result of the pooled specificity was 0.85 (95%CI = 0.80~0.88). The Chi-square value of the pooled sensitivity was 17.12 (P<0.05), and the I-square was 59.1%. (3C)The forest chart of the pooled positive likelihood ratio of CECT. The result of the pooled positive likelihood ratio was 5.00 (95%CI = 3.12~8.02). (3D)The forest chart of the pooled negative likelihood ratio of CECT. The result of the pooled negative likelihood ratio was 0.15 (95%CI = 0.10~0.21).

CECT: The results of the pooled sensitivity and pooled specificity and their 95%Cis were 0.90 (0.85~0.93) and 0.85 (0.80~0.88), respectively. The pooled positive likelihood ratio and the pooled negative likelihood ratio were 5.00 (3.12~8.02) and 0.15 (0.10~0.21), respectively. These results are shown in Fig 3.

**Fig 4:**
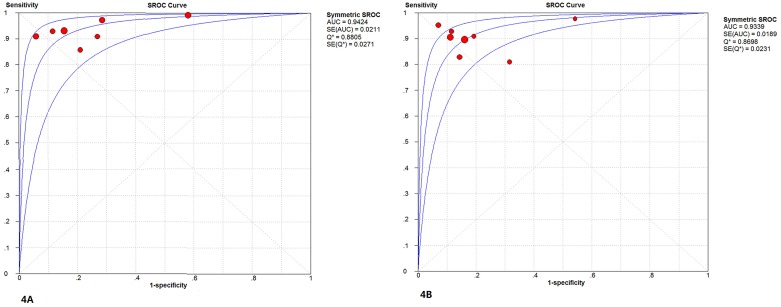
The SROC curve of CEUS and CECT. (4A)The AUC-SROC of CEUS was 94.24%, and the Q value was 0.8805. (4B)The AUC-SROC of CECT was 93.39%, and the Q value was 0.8698.

AUC-SROC: The AUC-SROC of the CEUS was 94.24% and the AUC-SROC of the CECT was 93.39% (Fig 4). The estimated Q values were 0.8805 and 0.8698, respectively. Comparing the Q index of the CEUS and CECT, we found no significant difference between the two methods (P>0.05).

#### 2.3 Sensitivity analysis

Measures of extreme variation and low methodological quality were recorded for some studies. The pooled sensitivity and specificity were calculated repeatedly after excluding each study sequentially, and no significant change was found. After removing the highest and lowest quality studies, the effect values remained within the 95%Cis of the original pooled sensitivity and specificity. This showed that the stability of the pooled results was acceptable. For example, consider the sensitivity analysis of the pooled specificity of CEUS. First, according to the forest plot, the results of Yong Xu et al. were markedly different from those of all of the other studies. Thus, this study was regarded as an outlier and was removed from the main analysis. A slight improvement was found in the pooled specificity, which was 0.84 (95%Cis 0.78, 0.88) after the removal of the outlier study; the pooled specificity of all of the studies was 0.79 (0.74~0.84). However, there was a slight decrease in the heterogeneity of the pooled specificity (Chi-square 11.66; P = 0.0397; I-square = 57.1%) compared with the pooled estimates of all of the studies (Chi-square 30.40 (P<0.001), I-square = 80.3%) in our meta-analysis.

#### 2.4 Meta-regression analysis

**Fig 5:**
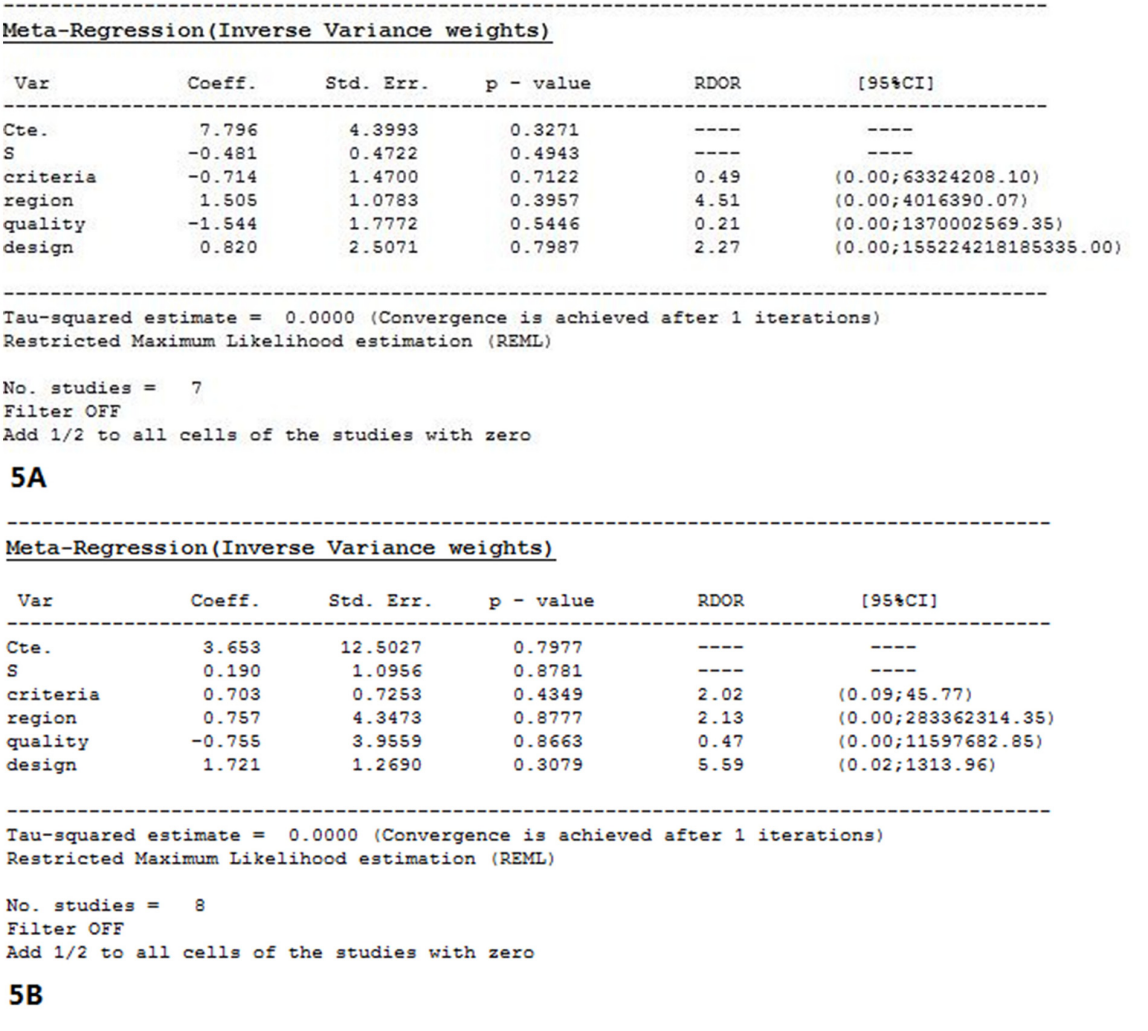
The meta-regression analysis of CEUS and CECT. (5A)The results indicate that the region, cases, design of the included studies related to CEUS, and choice of gold standard were not the main sources of heterogeneity. (5B)The results indicate that the region, cases, design of the included studies related to CECT, and choice of gold standard were not the main sources of heterogeneity.

The region, cases, study design and choice of the gold standard may cause heterogeneity in the independent variables in meta-regression analyses. The results indicated that these factors were not the main sources of heterogeneity, as shown in Fig 5.

## Discussion

Quality evaluation of the studies: Item 3 was a manuscript for which “no” was selected in the quality evaluation because the cases in this study were undetermined renal masses identified using CT, including cystic lesions and solid nodules. The final extracted data only included cystic lesions. For Item 6, “yes” was selected, indicating that the cases were identified exclusively by using the postoperative pathological results as the gold standard. “No” means that the cases in the manuscript were identified by both the postoperative pathological results and follow-up observation as the gold standard. For Item 12, the clinical interpretation bias was not illustrated in all of the studies. Some studies reported cases that were lost to follow up; the data from such studies were not included because of the absence of the final results. This assures that the selection of cases, the experimental evaluation method, the choice of gold standard and the implementation of the study design of the included articles are complete, thereby avoiding relevant bias as much as possible.

Both benign and malignant renal masses can be cystic in appearance, and surgical resection is the primary treatment method for malignant renal masses. Therefore, the timely and accurate diagnosis of complex renal cystic lesions is essential. Currently, the evaluation of the diagnostic results of the CECT, CEUS and CEMRI of renal cystic masses depends on the Bosniak classification, which is based on CECT features, such as the wall, number and thickness of septa; the presence of mural solid nodules; the enhancement of septa and nodules; and the calcifications, to stratify the malignant potentials of complicated renal cysts. Bosniak categories (I, II, IIF, III, and IV) suggest, in ascending order, the increasing probability of malignancy. The diagnoses of I and IV are generally clear, but opinions regarding the diagnosis of IIF and III remain divided [4]. Independently of the reason for not obtaining a conclusive diagnosis by CT, Therefore, we performed a meta-analysis to increase the statistical evidence underlying the Bosniak categories and used the most recent and relevant literature to increase sample quantity and reduce random error.

Data analysis and discussion: CECT is a traditional method used to diagnose malignant cystic lesions. An irregular thickened wall and septa and an abnormal enhancement of the intracystic solid components have been shown to constitute strong evidence for the prediction of malignancy [5]. The pooled sensitivity and specificity of CECT were 90% (95% CI, 0.85~0.93) and 85% (95% CI, 0.80~0.88), respectively. The pooled positive likelihood ratio and the pooled negative likelihood ratio were 5.00 and 0.15, respectively. The AUC-SROC was 93.39%. These data confirm the value of the diagnosis and differential diagnosis of CECT for renal cystic lesions. CEUS is a relatively new but increasingly utilized diagnostic modality. The results revealed that the pooled sensitivity and specificity of CEUS were 95% (95% CI, 0.91~0.98) and 79% (95% CI, 0.74~0.84), respectively. The pooled positive likelihood ratio and the pooled negative likelihood ratio were 4.39 and 0.10, respectively. The AUC-SROC was 94.24%. The correlation diagnostic evaluation parameter of CEUS was similar to that of CECT and the 95%Cis of the corresponding diagnostic parameters between CECT and CEUS are partially overlapped, which could explain why CEUS also has a high value for the diagnosis of renal cystic lesions. Because the CEUS standard in the diagnosis of cystic lesions is based on the Bosniak classification, this technique can be regarded as a supplement to CECT. The most important factor affecting the diagnosis of a cystic renal mass is the distribution of vascularization in the intracystic septa and intracystic nodules [6]. Some studies have suggested that CEUS may be superior to CECT for the observation of renal tumor micro vessels.

Our analyses revealed that the pooled sensitivity of CEUS was 95%, which is slightly higher than corresponding value of 90% for CECT, and that the pooled specificity of CEUS was 79%, which is slightly lower than the corresponding value of 85% for CECT. The differences in diagnostic accuracy between CECT and CEUS may result from different physical principles between them. The main components of the CECT contrast agent are small molecular particles, which can be dissolved in water and dispersed through the capillary wall. The delayed phase-enhanced images were performed on CT. In contrast, the microbubble, which is the main component of the CEUS contrast agent, cannot diffuse to the extracellular space as its diameter is larger and its size is similar to that of a red blood cell. However, it has a strong scattering property that enhances the echo intensity of blood, allowing small and even tiny capillaries to be displayed, which provides improved sensitivity in detecting blood flow. CEUS is extremely sensitive in revealing even the tiny capillaries that feed hairline septa, which allows CEUS images to depict more septa, thicker walls and septa, and more solid components in cystic renal lesions than can CECT[7,8,9]. The enhanced display of the distribution of vascularization in the intracystic septa and nodules in CEUS, which allows the detection of minimal contrast enhancement of septa, may fail in CT as CEUS is able to detect even very small amounts of microbubbles, whereas the partial volume effect of CECT may produce an indeterminate result. Additional information regarding the perfusion of the cystic septa or cystic renal cancer can be gained due to the real-time examination of CEUS[5,6].

For malignant lesions, the peripheral wall or septa and intracystic nodules mainly indicate intensive enhancement after contrast microbubbles are received. However, the microbubbles circulating in the septal vessels do not necessarily represent a malignancy as some minimal septal enhancement may also occur in benign renal cystic lesions. In case of benignity, multilocular cystic nephromas that revealed a peripheral wall with enhanced hairline-thin and thick septa can be easily misclassified as malignant after the review of CEUS examinations. Inflammatory or hemorrhagic cysts show a peripheral regular wall with enhancement and are not easily misdiagnosed because most do not reveal intracystic septations [10]. Data from several control studies have suggested that some lesions were shown to be Bosniak IIF based on CECT but Bosniak III or IV based on CEUS. The ultimate gold standard test usually confirmed that the lesion was malignant. This difference may be related to the higher sensitivity of CEUS in the diagnosis of renal cysts and indicates that CEUS may have an advantage over CECT for reducing missed diagnoses [11,12]. The Bosniak classification that base on the appearance of renal cysts at CT is accurate for predicting malignancy on CECT, either nodular or septal enhancement has shown diagnostic accuracy. Most renal cysts show no early enhancement in the corticomedullary phase and show washout in the parenchymal phase. The parenchymal phase shows greater septa enhancement compared with the corticomedullary phase. Delayed enhancement of the septa is likely due to the poor vascularity of renal cysts compared with normal kidney tissues [7,13].

Usually, sensitivity and specificity were inversely proportional that the sensitivity increased, specificity would decrease. The flow shown in hairline septa in CEUS can be a negative or positive indicator. It can be positive in that a lack of flow excludes a vascularized septa and largely excludes malignancy; however, it can be negative in that enhanced septa can be demonstrated in benign cysts. As a result, the specificity of enhancement in septa for malignancy is lower by CEUS than by CT. Therefore, diagnostic preference for high sensitivity or high specificity should ultimately be determined by the clinical outcome. However, we believe that high sensitivity is more beneficial than high specificity because better prognoses would be obtained as malignant cysts could be surgically treated earlier. In addition, mortality and morbidity are not significant with use of current surgical techniques related to the surgical resection of cysts [14,15].

Currently, CEUS technology is widely used in surgery. In the qualitative diagnosis of sentinel lymph node biopsies in breast cancer, a CEUS-guided biopsy can be more accurate and help to improve the diagnosis of the disease [16]. In the microwave ablation of hepatocellular carcinoma, CEUS can compensate for the defects of ordinary ultrasound in the positioning process of small lesions that are not clear [17]. Intracavitary CEUS can facilitate the determination of the correct setting of the drainage tube or needle in an abdominal or pelvic abscess drainage operation[18]. Studies concerning the use of CEUS in differentiating between benign and malignant renal solid lesions are currently being pursued [14,15]. Applications of CEUS for monitoring the minimally invasive treatments of renal masses including cryoablation (CA) and radiofrequency ablation (RFA) in both intra-procedural and post-procedural settings are also emerging. Real-time monitoring via CEUS is more advantageous than ultrasound and CT in positioning and qualitative analysis [19,20]. The quantitative analysis of renal masses using CEUS is another promising area of research. Some researchers have confirmed that early washout in the area of maximal intensity in the interior of the lesion and prolonged washout in the whole area of the lesion are specific manifestations in CEUS that suggest RCC [21].

The heterogeneity of the data and limitations of this study are as follows. (1) Although the search strategy combined key words and manual retrieval, it is possible that relevant literature was missed because of incomplete retrieval. (2) This study excluded experience exchanges, abstracts, lectures, reviews, conference papers, and other secondary literature during retrieval, and these articles may contain high quality research. (3) The Bosniak classification system has some subjectivity. The experience, knowledge and other characteristics of the imaging experts are likely to have impacts on the classification results. (4) Patients in the study came from different countries and regions. Racial differences include differences in heredity and living environment, which may have unknown impacts on the stages of the disease. (5) The principle of informed consent must be followed in the actual clinical diagnosis and treatment. Therefore, it is difficult to achieve a completely randomized controlled trial that will produce a particular heterogeneity. (6) Most cases were selected from the incidental findings on grey US. Grey US is usually regarded as a means of physical examination screening, and its diagnostic value is limited because lesions with a minimal nodule or solid component may be difficult to definitively diagnose by US but are visible on conventional CT or MRI. The lack of comparative morphologic criteria between US and CT and the selection of enrolled cases that were based on US findings may be additional study limitations. (7) The evaluation of the CT methodology varied among the included studies. Some CT studies only reviewed axial CT images in the absence of coronal or sagittal images, whereas the wall and septa of complicated renal cysts may have displayed better on multiplanar reformatted images in some patients. In addition, the CT examinations were performed using a variety of single-detector CT, double-detector CT and multi-detector CT (MDCT) scanners with different scanning parameters. MDCT is performed using thin collimation scanning, which can potentially improve the capability of CT in displaying the enhanced components. Furthermore, the slice thickness differed according to the channel number of the MDCT scanners, and the use of tube current in each patient varied because of the automatic dose modulation technique. This variability may cause inconsistent determination of the septa and wall features. However, this variability was inevitable because it requires a long time to collect a sufficient number of cases from one institution due to the low morbidity rate of cystic RCCs. And it was the inherent limitations of these retrospective studies.

**Fig 6:**
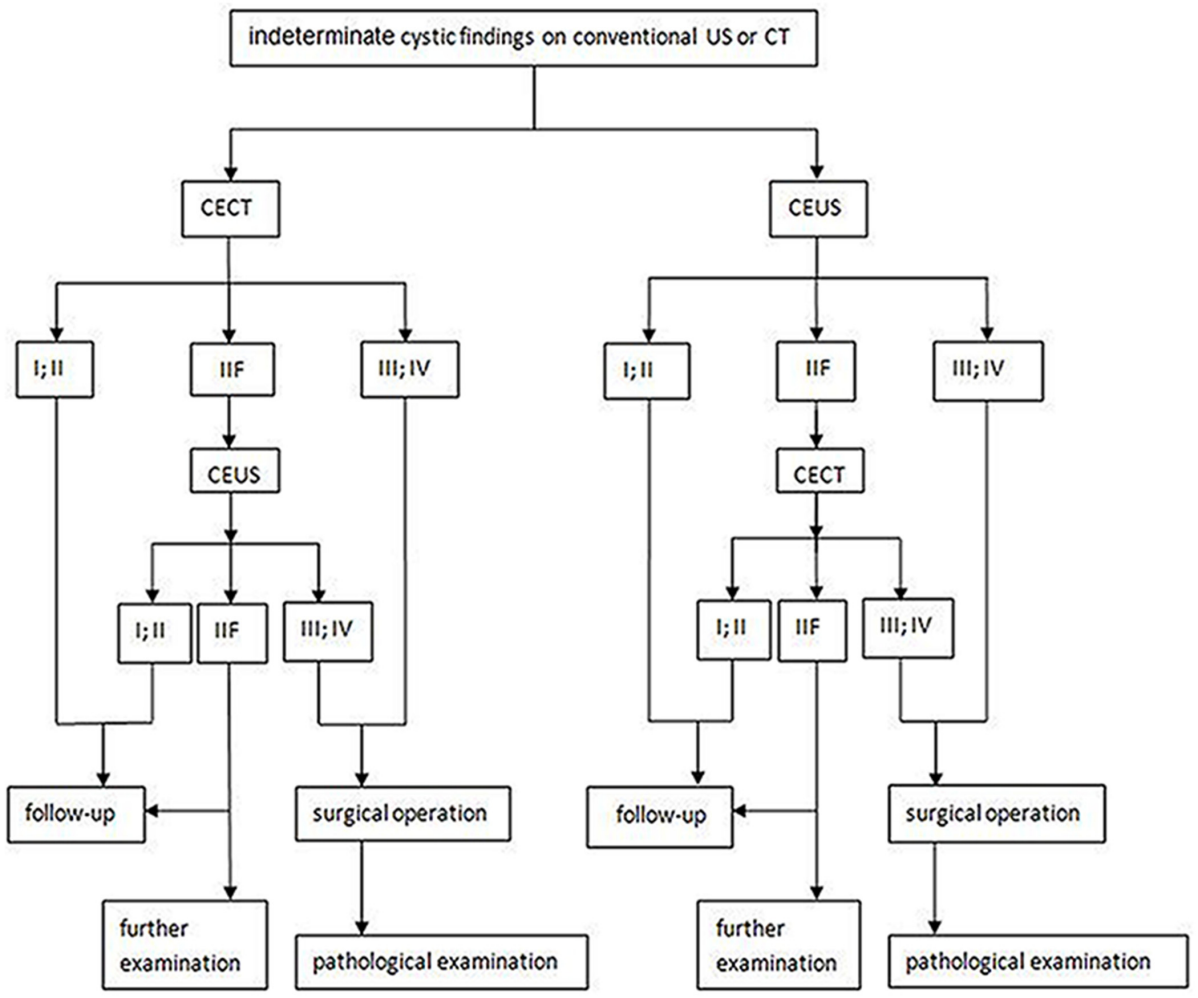
Diagnostic flow chart. A diagnostic flow chart for indeterminate lesions based on the results of the meta-analysis related to CEUS and CECT.

The aim of this meta-analysis was to confirm whether CEUS is useful in evaluating the management of indeterminate lesions and identifying lesions with features. we give a diagnostic flow chart (Fig 6) for indeterminate lesions based on the results of the meta-analysis. Several studies have reported that CEUS has other advantages over CECT: safe contrast agents, with a very low incidence of side effects including allergic reaction (the allergic skin test is nonessential before injection); the absence of X-ray radiation; continuous real-time dynamic imaging; a low risk of adverse reactions to the contrast agent; and no hepatotoxicity or nephrotoxicity of the contrast agent (the contrast agent of CECT needs to be metabolized by the liver or kidney before excretion, whereas the contrast microbubbles of CEUS are excreted through the lungs). As a result, this technique is equally applicable for patients with hepatic and renal insufficiencies [22]. Diffusion weighted MRI, another non-invasive modality with no risk of radiation and can be used in patients with deranged renal failure. However, its expense and time requirement and the potential nephrotoxicity of contrast agent have limited its use. Israel [23]found that in comparison with CT, MRI depicted additional septa, thickening of the wall and/or septa, or even enhancement in some cases. Such MRI results may lead to an upgrade of the Bosniak classification and have possible effects on patient management. On the other hand, Diffusion weighted MRI, other type of imaging technology with a different theoretical basis, is equally worthy of careful study before we evaluate its diagnostic value in this field.

## Conclusions

This study suggests that the value of CEUS in the diagnosis of malignant renal cystic lesions is similar to that of traditional CECT, which confirms that CEUS is a very useful tool for characterizing complex renal cysts. CEUS may be very useful for further characterizing renal lesions with indeterminate enhancement on CT because of its improved visualization of tumor microvessels.

However, we recommend that these two imaging technologies be used in combination because our results showed that CEUS has higher pooled sensitivity than does CECT, whereas CECT has higher pooled specificity than CEUS; therefore, their combined use may both improve the lesion detection rate and decrease the misdiagnosis rate. CEUS is a safe, simple, and highly repeatable examination method, and it has broad prospects for future development and application. Given that most of the literature included in this study was retrospective, case-selection bias may have occurred because some cases may have not been included. Prospective randomized controlled trials with larger samples will be needed to confirm our results in the future.

## Supporting Information

S1 PRISMA Checklist The items for a systematic review or meta-analysis. (DOC)
